# ChatGPT provides accurate and safe responses to patient questions on hip arthroscopy, while completeness remains variable: A systematic review and single‐arm meta‐analysis

**DOI:** 10.1002/ksa.70396

**Published:** 2026-04-14

**Authors:** Nikolai Ramadanov, Plamen Penchev, Maximilian Voss, Maximilian Heinz, Robert Prill, Mikhail Salzmann, Roland Becker, Timoty Osterberger, Ingo J. Banke

**Affiliations:** ^1^ Center of Orthopaedics and Traumatology, Brandenburg Medical School University Hospital Brandenburg an der Havel Brandenburg an der Havel Germany; ^2^ Faculty of Health Science Brandenburg, Brandenburg Medical School Theodor Fontane Brandenburg an der Havel Germany; ^3^ Faculty of Medicine Medical University of Plovdiv Plovdiv Bulgaria; ^4^ Clinic of Orthopaedics and Sports Orthopaedics, School of Medicine and Health TUM University Hospital, Technical University of Munich Munich Germany; ^5^ AGA‐Society for Arthroscopy and Joint‐Surgery, Hip Committee, c/o Walder Wyss Ltd. Zürich Switzerland

**Keywords:** accuracy, ChatGPT, completeness, femoroacetabular impingement, hip arthroscopy, meta‐analysis

## Abstract

**Purpose:**

Large language models, such as ChatGPT, are increasingly used by patients seeking information on hip arthroscopy (HAS) and femoroacetabular impingement (FAI). Despite their linguistic fluency, the accuracy, completeness and safety of procedure‐specific patient information remain unclear. Although orthopaedic studies report variable performance across subspecialties, no systematic evaluation has specifically addressed HAS.

**Methods:**

PubMed, Embase, Scopus, CINAHL and Epistemonikos were searched to 10 February 2026 for studies evaluating ChatGPT responses to patient‐oriented HAS or FAI questions. Randomized and non‐randomized studies, observational cohorts and case series were eligible. Data on question sources, model versions, rating systems and performance domains (accuracy, relevance, completeness, safety, readability and clarity) were extracted. Heterogeneous rating scales were dichotomized into high‐ versus low‐quality responses. Risk of bias was assessed using QUADAS‐2 and ROBINS‐I. Random‐effects single‐arm meta‐analyses (REML) were conducted for each domain.

**Results:**

Eight studies met eligibility criteria. Accuracy was high (pooled 88.6%). Relevance, safety, readability and clarity reached pooled values of 100% with low heterogeneity. Completeness was lower (83.8%) with moderate heterogeneity, mainly driven by early GPT‐3.5 studies. Funnel plots showed no clear small‐study effects, although interpretation was limited by the small number of studies. Risk of bias was predominantly high or moderate, largely due to non‐systematic question selection and heterogeneous rating tools. Later models (GPT‐4/4o and beyond) demonstrated higher performance compared with GPT‐3.5.

**Conclusion:**

ChatGPT provides accurate, relevant, safe and clear responses to patient questions about HAS, while completeness shows moderate variability. Although LLMs appear promising as adjuncts to patient education, methodological limitations in the current evidence base underscore the need for expert clinical counselling and more rigorous, standardized evaluation frameworks.

**Level of Evidence:**

Level III systematic review and meta‐analysis of non‐randomized studies.

**Abbreviations**
AI: artificial intelligence
CI: confidence interval
FAI: femoroacetabular impingement
HAS: hip arthroscopy
*I* ^2^
: Higgins' heterogeneity statistic
LLM: large language model
PRISMA: Preferred Reporting Items for Systematic Reviews and Meta‐Analyses
PROSPERO: International Prospective Register of Systematic Reviews
QUADAS‐2: Quality Assessment of Diagnostic Accuracy Studies–2
REML: restricted maximum likelihood
ROBINS‐I: Risk Of Bias In Non‐randomized Studies of Interventions

## INTRODUCTION

Large language models (LLMs) such as ChatGPT are increasingly used by patients to obtain information about orthopaedic conditions and surgical procedures. Despite their accessibility and linguistic fluency, concerns remain regarding the reliability, completeness, and safety of artificial intelligence (AI)‐generated medical information.

Previous orthopaedic studies have demonstrated generally acceptable performance of ChatGPT in subspecialties such as meniscus surgery and anterior cruciate ligament reconstruction, although variability and omission of clinically relevant details have been reported [13, 25, 28]. Moreover, AI‐generated responses are frequently perceived as highly credible and empathic, sometimes equalling or exceeding surgeon‐provided explanations in believability [12, 15, 19], which may mask incomplete or oversimplified information.

Hip arthroscopy (HAS) represents a particularly relevant context, as it is an elective procedure performed in a young, digitally engaged patient population in whom pre‐consultation online information may substantially influence expectations, risk perception and shared decision‐making.

However, ChatGPT's performance has not yet been systematically evaluated specifically for HAS. Given the procedure‐specific nuances regarding indications, intra‐articular pathology and post‐operative rehabilitation, extrapolation from other orthopaedic subspecialties may not be appropriate.

The purpose of this study was to systematically synthesize available evidence on ChatGPT's performance in answering patient‐oriented questions related to HAS. It was hypothesized that ChatGPT would demonstrate high accuracy and safety but variable completeness and clinical specificity, particularly in earlier model generations.

## METHODS

### Reporting framework and registration

The protocol was prospectively registered in PROSPERO (CRD420251181100; 31 October 2025). The review followed updated Preferred Reporting Items for Systematic Reviews and Meta‐Analyses (PRISMA) guidelines [17]. The completed PRISMA checklist is provided in Appendix S1.

### Search strategy

PubMed, Embase, Scopus and Epistemonikos were searched from inception to 10 February 2026. Search terms combined controlled vocabulary and free‐text terms related to ChatGPT/LLMs and HAS/femoroacetabular impingement (FAI). No restrictions on publication year or language were applied.

### Study selection

Two reviewers (NR and MV) independently screened titles/abstracts and full texts. Discrepancies were resolved by a third reviewer (IJB). Inter‐rater reliability was assessed using Cohen's *κ*.

### Eligibility criteria

Randomized trials, comparative studies, observational studies and case series evaluating ChatGPT responses to patient‐oriented questions on HAS or FAI were included. Case reports, narrative reviews, commentaries and editorials were excluded.

### Data extraction

Extracted data included: study characteristics, ChatGPT model version, number and background of reviewers, number and source of patient questions, evaluation domains, rating scales and domain‐specific outcomes. For each domain, we recorded the number of high‐quality responses and the total evaluated items. When binary outcome data were not available, authors were contacted for clarification.

### Outcome transformation

Because rating systems varied across studies, outcomes were dichotomized into high‐quality (upper performance category as defined by the original study) and low‐quality responses. For each domain, the number of high‐quality responses and total ratings were extracted. Only studies reporting a specific domain were included in that domain's analysis (Table 1).

**Table 1:** Operationalisation of rating scales and binary definition of ‘high‐quality’ ChatGPT responses.

Scale type	Scale levels	Definition of ‘acceptable’
4‐Point Ordinal Rating Scale (Mika et al. system)	1 = Excellent 2 = Satisfactory – minimal clarification 3 = Satisfactory – moderate clarification 4 = Unsatisfactory	1 or 2 = acceptable
Letter grade (A–D)	A = Excellent B = Good C = Fair D = Poor	A or B = acceptable
5‐point Likert	1 = Very poor 2 = Poor 3 = Fair 4 = Good 5 = Excellent	4 or 5 = acceptable
4‐tier categorical	Excellent Minimal clarification Moderate clarification Poor	Excellent or Minimal clarification = acceptable

### Performance domains

The following domains were evaluated: (1) Accuracy: factual correctness [5], (2) Relevance: directly addressing the question [21], (3) Completeness: coverage of essential information [5], (4) Safety: absence of harmful or misleading content [5], (5) Readability: layperson accessibility [5] and (6) Clarity: logical and unambiguous presentation [21].

### Quality assessment

Risk of bias was assessed independently by two reviewers using QUADAS‐2 [27] and ROBINS‐I [24]. For QUADAS‐2, ChatGPT output was considered the index test and expert ratings the reference standard. Disagreements were resolved by consensus.

### Statistical analysis

Single‐arm random‐effects meta‐analyses [11] were performed to estimate pooled proportions of high‐quality responses per domain. Binary data were transformed using the Freeman–Tukey double‐arcsine method, and pooled proportions with 95% confidence intervals (CIs) were calculated using REML estimation [20]. Heterogeneity was assessed using *I* ^2^. Funnel plots evaluated publication bias. Analyses were conducted in R (meta, metafor). Sensitivity analyses were performed by excluding outlier data sets to assess robustness.

## RESULTS

### Systematic search results

**Figure 1:**
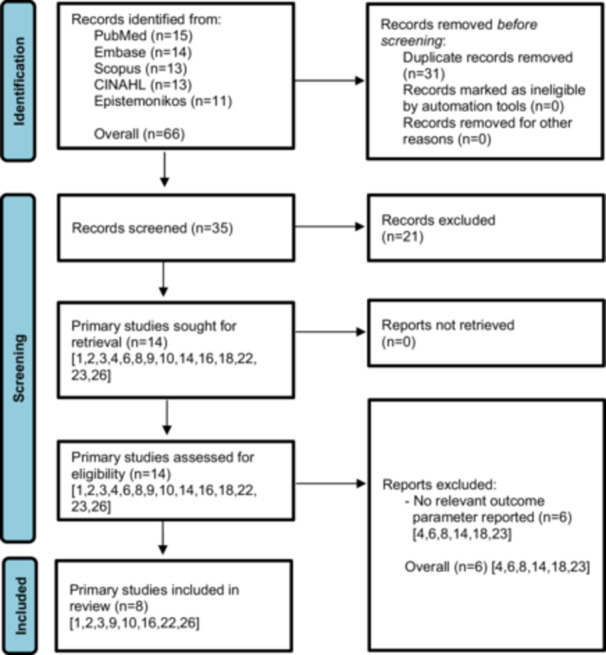
PRISMA flow diagram of the study selection process. The search yielded 66 records, of which 31 duplicates were removed. After screening 35 titles and abstracts, 14 full‐text reports were assessed for eligibility. Six were excluded [4, 6, 8, 14, 18, 23] for not reporting relevant outcomes, resulting in eight primary studies included in the final review [1, 2, 3, 9, 10, 16, 22, 26]. PRISMA, Preferred Reporting Items for Systematic Reviews and Meta‐Analyses.

The database search (PubMed, Embase, Scopus, CINAHL and Epistemonikos) identified 66 records. After removal of 31 duplicates, 35 titles and abstracts were screened (*κ* = 1.0). Fourteen studies [1, 2, 3, 4, 6, 8, 9, 10, 14, 16, 18, 22, 23, 26] underwent full‐text assessment (*κ* = 1.0); six [4, 6, 8, 14, 18, 23] were excluded for not reporting relevant outcomes. Eight primary studies [1, 2, 3, 9, 10, 16, 22, 26] met the eligibility criteria and were included in the meta‐analysis (Figure 1).

### Descriptive results

Eight studies [1, 2, 3, 9, 10, 16, 22, 26] evaluated different ChatGPT versions (GPT‐3.5 to GPT‐5.0) in answering patient questions on HAS and FAI (Table 2). Sample sizes ranged from 10 to 30 questions, assessed by 2–10 independent raters. Question sources included hospital FAQs, search engines, social media and model‐generated prompts. For one study [9], dichotomized data were obtained directly from the authors.

**Table 2:** Characteristics of included studies [1, 2, 3, 9, 10, 16, 22, 26] evaluating ChatGPT responses to hip arthroscopy‐related patient questions.

Study/first author	Journal	ISSN	Country	Model version	Rater background	Question source	Domains rated	Scale type
Adelstein et al. [1]	*Clinical Journal of Sport Medicine*	1536‐3724	USA	GPT‐3.5	Two orthopaedic surgery resident authors	Patient FAQs from Yale, Cleveland Clinic, University of Washington, HSS, Twin Cities	Accuracy	4‐Point Ordinal Rating Scale (Mika et al. system)
AlShehri et al. [2]	*Arthroscopy: The Journal of Arthroscopic and Related Surgery*	1526‐3231	Saudi Arabia	GPT‐3.5	Two orthopaedic surgeons (fellowship‐trained hip arthroscopy)	Google Trends + social media (Reddit, Quora)	Relevance, Accuracy, Completeness, Readability, Safety	Letter grade (A–D)
Ayik et al. [3]	*Joint Diseases and Related Surgery*	2687‐5951	Turkiye	GPT‐4o	Ten orthopaedic surgeons specializing in sports surgery	Google search + PubMed patient education pages + hospital FAQs	Relevance, Accuracy, Clarity, Completeness	5‐point Likert
Gültekin et al. [9]	*Knee Surgery, Sports Traumatology, Arthroscopy*	1433‐7347	Turkiye	GPT‐4o	Two fellowship‐trained orthopaedic surgeons	Reputable orthopaedic health information websites	Accuracy, Completeness, Readability, Clarity	5‐point Likert
Gültekin et al. [9]	*Knee Surgery, Sports Traumatology, Arthroscopy*	1433‐7347	Turkiye	GPT‐4o + Deep Research	Two fellowship‐trained orthopaedic surgeons	Reputable orthopaedic health information websites	Accuracy, Completeness, Readability, Clarity	5‐point Likert
Heinz et al. [10]	*Medicina*	1648‐9144	Germany	GPT‐3.5	Two independent fellowship‐trained orthopaedic surgeons	Auto‐generated by ChatGPT‐3.5	Accuracy, Relevance, Completeness, Clarity	5‐point Likert
Ozbek et al. [16]	*Arthroscopy: The Journal of Arthroscopic and Related Surgery*	1526‐3231	Turkiye	GPT‐4.0	Two orthopaedic sports medicine surgeons	Health forums + Google search + social media	Relevance, Accuracy, Safety	4‐tier categorical
Slawaska‐Eng et al. [22]	*Journal of ISAKOS*	2059‐7762	United Kingdom	GPT‐3.5	Three fellowship‐trained, high‐volume hip arthroscopy surgeons	Standardized patient FAQ set from NHS + Google search	Accuracy, Safety, Factual Correctness	4‐tier categorical
Slawaska‐Eng et al. [22]	*Journal of ISAKOS*	2059‐7762	United Kingdom	GPT‐4.0	Three fellowship‐trained, high‐volume hip arthroscopy surgeons	Standardized patient FAQ set from NHS + Google search	Accuracy, Safety, Factual Correctness	4‐tier categorical
Voss et al. [26]	*Indian Journal of Orthopaedics*	2077‐0383	Germany	GPT‐5.0	Two independent fellowship‐trained orthopaedic surgeons	Auto‐generated by ChatGPT‐5.0	Accuracy, Relevance, Completeness, Clarity	5‐point Likert

Accuracy was reported in all studies and ranged from 7/10 in early GPT‐3.5 evaluations to 25/25 in later GPT‐4/5 assessments. Relevance, clarity and completeness were assessed in most studies and generally demonstrated high ratings. Safety was evaluated in three studies and consistently rated high, whereas readability was reported only once (Table 3).

**Table 3:** Number of high‐quality ChatGPT responses per evaluation domain and study.

Study ID	Scale type	Number of raters	Number of questions	Accuracy	Relevance	Completeness	Safety	Readability	Clarity
Adelstein et al. [1]	4‐Point Ordinal Rating Scale (Mika et al. system)	2	10	7	NR	NR	NR	NR	NR
AlShehri et al. [2]	Letter grade (A–D)	2	10	8	NR	5	NR	NR	NR
Ayik et al. [3]	5‐point Likert	10	20	20	20	20	20	20	20
Gültekin et al. [9]	5‐point Likert	2	30	19	NR	16	NR	18	13
Gültekin et al. [9]	5‐point Likert	2	30	16	NR	20	NR	15	23
Heinz et al. [10]	5‐point Likert	2	20	20	20	19	NR	NR	20
Ozbek et al. [16]	4‐tier categorical	2	25	25	25	23	25	NR	NR
Slawaska‐Eng et al. [22]	4‐tier categorical	3	12	9	12	9	12	12	NR
Slawaska‐Eng et al. [22]	4‐tier categorical	3	12	11	12	11	12	12	NR
Voss et al. [26]	5‐point Likert	2	25	25	25	24	NR	NR	25

Abbreviation: NR, not reported.

Most post‐2024 models (GPT‐4o and later) showed near‐ceiling performance across accuracy, relevance, clarity, and completeness, suggesting generational improvement compared with GPT‐3.5 models. However, one data set [9] reported substantially lower proportions across multiple domains, contributing to between‐study heterogeneity.

### Quality assessment

QUADAS‐2 revealed a high risk of bias in the question selection domain across all studies due to reliance on author‐selected or model‐generated questions. The index test and flow/timing domains were generally low risk. The reference standard domain showed variability, with non‐validated rating tools and limited inter‐rater reliability in several studies. Overall, all studies were classified as high risk of bias under QUADAS‐2 (Table 4).

**Table 4:** Risk of bias of included studies according to the adapted QUADAS‐2 framework.

Study	Patient/question selection	Index test	Reference standard	Flow and timing	Overall risk
Adelstein et al. [1]	High	Low	High	Low	High
AlShehri et al. [2]	High	Low	High	Low	High
Ayık et al. [3]	High	Low	High	Low	High
Gültekin et al. [9]	High	Moderate	Moderate	Low	High
Heinz et al. [10]	High	Low	Moderate	Low	High
Özbek et al. [16]	High	Low	Low	Low	High
Slawaska‐Eng et al. [22]	High	Low	Low	Low	High
Voss et al. [26]	High	Low	Moderate	Low	High

*Note*: All studies showed a high risk of bias due to non‐systematic question selection. The index‐test and flow‐and‐timing domains were consistently low risk. Variation in the reference‐standard domain—mainly driven by non‐validated rating scales or limited inter‐rater agreement—contributed to the overall high‐risk judgement.

ROBINS‐I indicated overall moderate risk of bias across studies, primarily driven by non‐systematic question selection and heterogeneity in rating methodology. Other domains, including intervention classification and missing data, were generally low risk (Table 5).

**Table 5:** ROBINS‐I risk of bias assessment of the included studies evaluating ChatGPT‐generated patient information on femoroacetabular impingement and hip arthroscopy.

Study	Confounding	Selection of participants (questions)	Classification of intervention	Deviations from intended intervention	Missing data	Measurement of outcomes	Selection of reported results	Overall risk of bias
Adelstein et al. [1]	Moderate	Moderate	Low	Low	Low	Moderate	Low	Moderate
AlShehri et al. [2]	Moderate	Moderate	Low	Low	Low	Moderate	Low	Moderate
Ayık et al. [3]	Low	Moderate	Low	Low	Low	Low	Low	Moderate
Gültekin et al. [9]	Moderate	Moderate	Low	Low	Low	Moderate	Low	Moderate
Heinz et al. [10]	Low	Moderate	Low	Low	Low	Low	Low	Moderate
Özbek et al. [16]	Low	Moderate	Low	Low	Low	Moderate	Low	Moderate
Slawaska‐Eng et al. [22]	Low	Moderate	Low	Low	Low	Moderate	Low	Moderate
Voss et al. [26]	Low	Moderate	Low	Low	Low	Low	Low	Moderate

*Note*: Each study was assessed across eight ROBINS‐I domains (confounding, selection of participants, classification of intervention, deviations from intended intervention, missing data, measurement of outcomes and selection of reported results). Domain‐level ratings are presented as low, moderate, serious or critical risk of bias. Overall risk of bias was determined by the highest domain rating for each study.

### Single‐arm meta‐analysis

#### Accuracy

**Figure 2:**
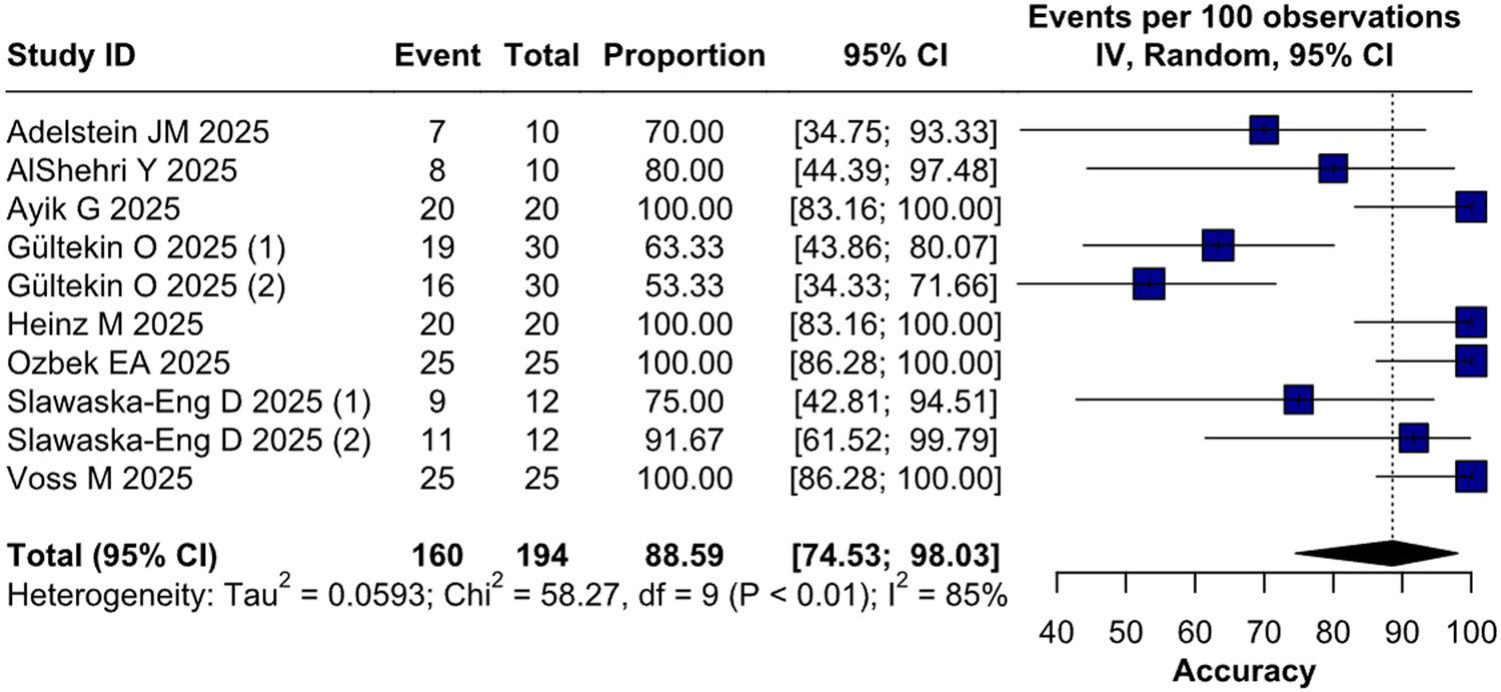
Forest plot of study‐level and pooled accuracy proportions for ChatGPT responses to hip arthroscopy questions. Accuracy ranged from 70% to 100%, with a pooled value of 88.6% (95% CI = 74.5–98.0). Heterogeneity was high (*I* ^2^ = 85%). CI, confidence interval.

Accuracy was high across studies, with a pooled estimate of 88.6% (95% CI = 74.5–98.0). Between‐study heterogeneity was substantial (*I* ^2^ = 85%) (Figure 2). The funnel plot is provided in Figure S1.

#### Relevance

**Figure 3:**
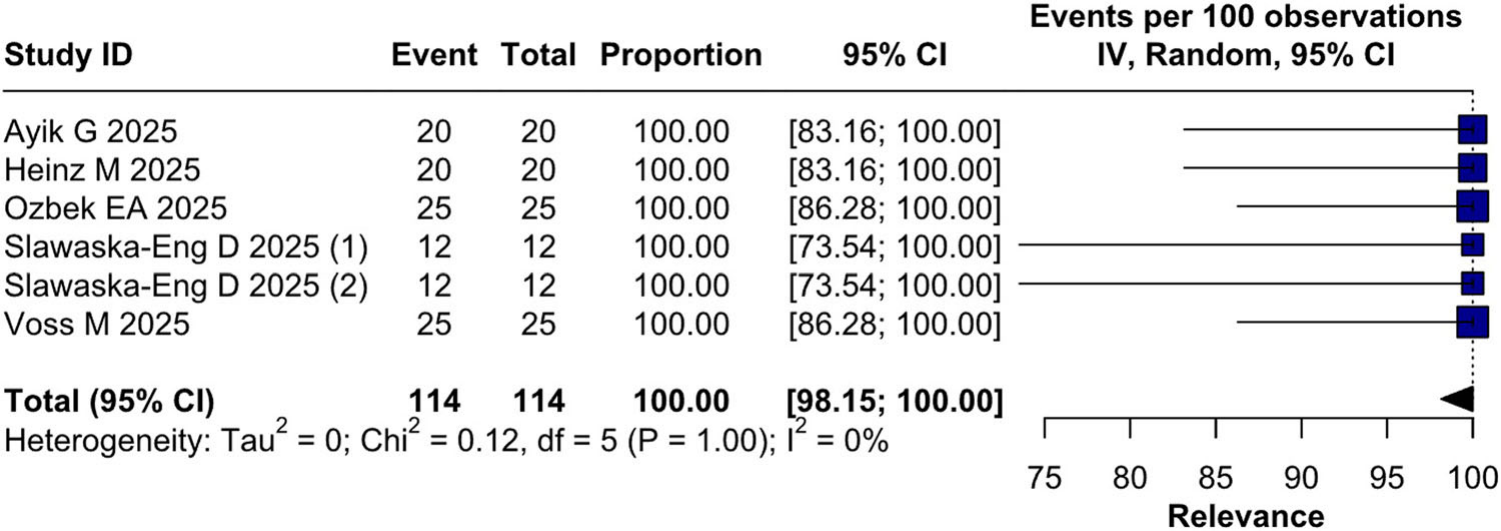
Forest plot of relevance proportions. All studies reported 100% relevance, resulting in a pooled estimate of 100% (95% CI = 98.2–100.0) with no heterogeneity (*I* ^2^ = 0%). CI, confidence interval.

All included studies rated responses as relevant, resulting in a pooled proportion of 100% (95% CI = 98.2–100.0) with no heterogeneity (*I* ^2^ = 0%) (Figure 3). The funnel plot is provided in Figure S2.

#### Completeness

**Figure 4:**
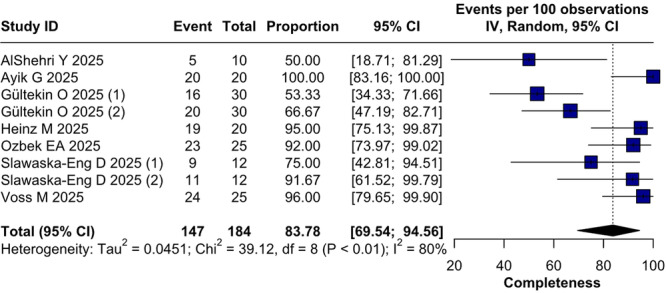
Forest plot of completeness proportions. Completeness ranged widely from 50% to 100%, with a pooled value of 83.8% (95% CI = 69.5–94.6). Heterogeneity was high (*I* ^2^ = 80%). CI, confidence interval.

Completeness demonstrated greater variability, with a pooled estimate of 83.8% (95% CI = 69.5–94.6) and high heterogeneity (*I* ^2^ = 80%) (Figure 4). Lower values were primarily driven by early GPT‐3.5 evaluations. The funnel plot is provided in Figure S3.

#### Safety

**Figure 5:**
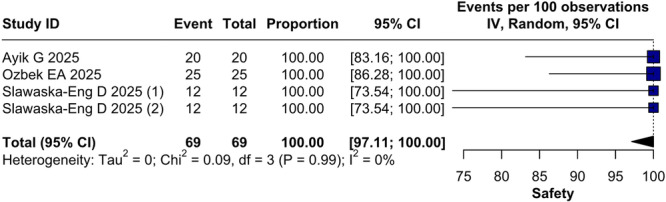
Forest plot of safety proportions. All studies reported 100% safety, producing a pooled value of 100% (95% CI = 97.1–100.0), with no heterogeneity. CI, confidence interval.

All contributing studies reported 100% safety, yielding a pooled estimate of 100% (95% CI = 97.1–100.0) without heterogeneity (*I* ^2^ = 0%) (Figure 5). The funnel plot is provided in Figure S4.

#### Readability

**Figure 6:**
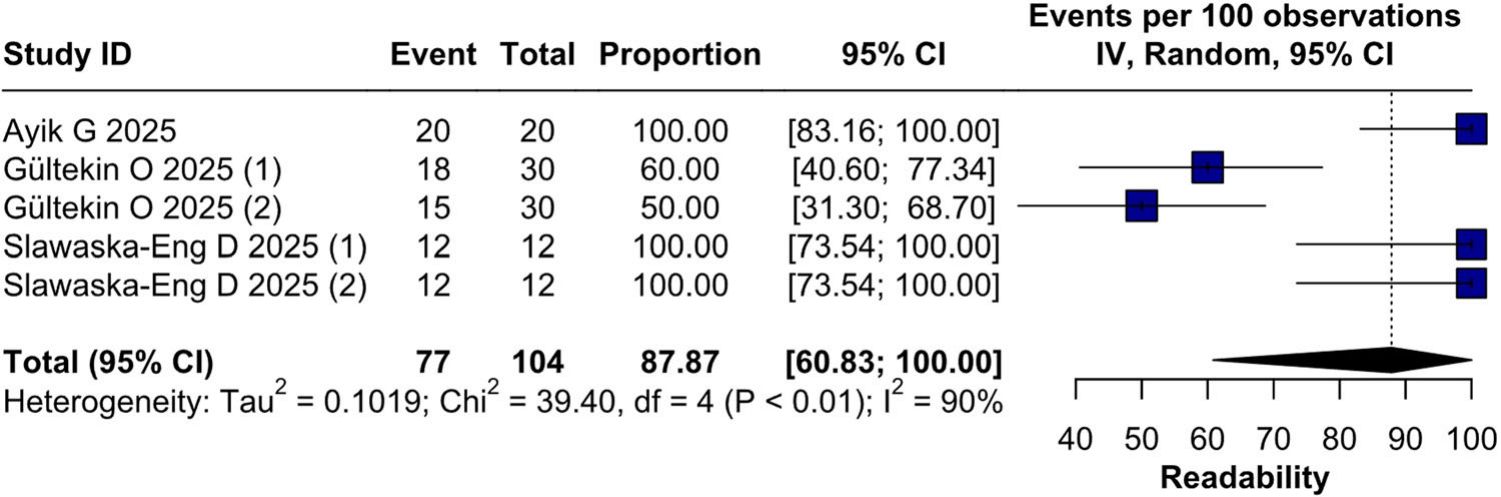
Forest plot of readability proportions. Readability ranged widely from 50% to 100%, with a pooled value of 87.9% (95% CI = 60.8–94.6). Heterogeneity was high (*I* ^2^ = 90%). CI, confidence interval.

Readability showed variability across studies, with a pooled estimate of 87.9% (95% CI = 60.8–100.0) and substantial heterogeneity (*I* ^2^ = 90%) (Figure 6). The funnel plot is provided in Figure S5.

#### Clarity

**Figure 7:**
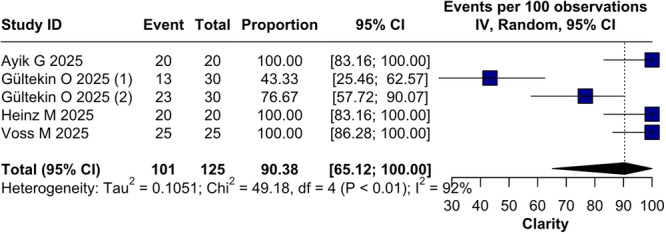
Forest plot of clarity proportions. Clarity ranged widely from 43% to 100%, with a pooled value of 90.4% (95% CI = 65.1–100.0). Heterogeneity was high (*I* ^2^ = 92%). CI, confidence interval.

Clarity was high overall, with a pooled estimate of 90.4% (95% CI = 65.1–100.0) and considerable heterogeneity (*I* ^2^ = 92%) (Figure 7). The funnel plot is provided in Figure S6.

#### Sensitivity analysis

Exclusion of Gültekin et al. [9] increased pooled proportions across several domains and markedly reduced heterogeneity, with *I* ^2^ decreasing to 0% in selected analyses (Figures [Link], [Link], [Link], [Link], [Link], [Link]). The direction and magnitude of pooled estimates remained consistent, confirming the robustness of the primary findings.

## DISCUSSION

The most important finding of the present study was that ChatGPT provides highly accurate, relevant and safe responses to patient‐oriented questions on HAS, while completeness remains more variable, particularly in earlier model generations. Across domains, pooled proportions frequently approached 100%, although interpretation must consider methodological limitations of the underlying studies.

Accuracy exceeded 88%, consistent with findings from other orthopaedic subspecialties [15, 25, 28]. Relevance reached 100%, reflecting strong subject–question alignment. In the only comparative trial, ChatGPT outperformed conventional web search results in overall quality and citation use, although readability demands remained similarly high [8].

Completeness demonstrated greater variability. One early GPT‐3.5 study reported substantially lower values, contributing to heterogeneity, whereas later GPT‐4 and subsequent models showed consistently improved completeness, suggesting generational advancements in informational depth. Safety was uniformly rated high; however, evaluations relied on study‐specific rating frameworks and did not include high‐risk or ambiguous clinical scenarios.

Readability and clarity were assessed in five studies and generally achieved high pooled values (~90%), although heterogeneity was influenced by a single data set. Prior evidence indicates that LLM outputs often exceed recommended reading levels [7], highlighting the need for standardized readability metrics in future HAS‐specific research.

Several limitations must be acknowledged. First, question selection represented a uniformly high risk of bias, as most studies relied on author‐selected or search‐derived FAQs rather than systematically collected patient‐generated questions. Second, variability in rating instruments and the necessary dichotomization of ordinal scales may have inflated pooled proportions and contributed to ceiling effects. Third, the small number of included studies per domain limits the robustness of pooled estimates. Fourth, all studies evaluated English‐language prompts, restricting generalizability to non–English‐speaking populations. Finally, safety assessments did not incorporate complex or high‐risk clinical scenarios, which may underestimate potential limitations of LLM outputs in real‐world consultations.

Despite these constraints, later model generations consistently demonstrated improved performance, supporting a temporal advancement trend observed in orthopaedic LLM research [7, 12, 13, 15, 19, 25, 28].

From a clinical perspective, these findings suggest that ChatGPT may provide reliable background information for patients seeking pre‐consultation knowledge on HAS. However, variability in completeness and methodological constraints preclude its use as a substitute for individualized clinical counselling or shared decision‐making. Awareness of both the strengths and limitations of AI‐generated information may enable orthopaedic surgeons to better contextualize, verify and supplement such content during patient interactions.

## CONCLUSION

ChatGPT demonstrates high accuracy, relevance, safety, clarity and readability in answering patient‐oriented questions on HAS, although completeness varies in earlier model generations. However, the current evidence is limited by non‐systematic question selection, heterogeneous rating tools and small sample sizes. ChatGPT may complement patient education but cannot replace individualized counselling or shared decision‐making. Future studies should evaluate responses to spontaneously generated patient questions to better reflect real‐world information needs.

## AUTHOR CONTRIBUTIONS

Nikolai Ramadanov and Maximilian Voss performed the literature search, the data extraction and the risk of bias assessment. Plamen Penchev and Nikolai Ramadanov conducted the statistical calculations. Plamen Penchev and Nikolai Ramadanov created all figures and tables. Nikolai Ramadanov wrote the manuscript. Ingo J. Banke, Robert Prill, Timoty Osterberger, Mikhail Salzmann, Maximilian Voss, Maximilian Heinz and Roland Becker supervised the work.

## CONFLICT OF INTEREST STATEMENT

The authors declare no conflicts of interest.

## ETHICS STATEMENT

The authors have nothing to report.

## Supporting information

Supporting information.

Supporting information.

Supporting information.

Supporting information.

Supporting information.

Supporting information.

Supporting information.

Supporting information.

Supporting information.

Supporting information.

Supporting information.

Supporting information.

Supporting information.

Supporting information.

## Data Availability

Available from the corresponding author upon reasonable request.
