# Supplementary figures and images for: ChatGPT provides accurate and safe responses to patient questions on hip arthroscopy, while completeness remains variable: A systematic review and single‐arm meta‐analysis
Source: Knee Surg Sports Traumatol Arthrosc. 2026 Apr 14;34(6):2236–46. doi: 10.1002/ksa.70396 (PMC13266954; doi:10.1002/ksa.70396)

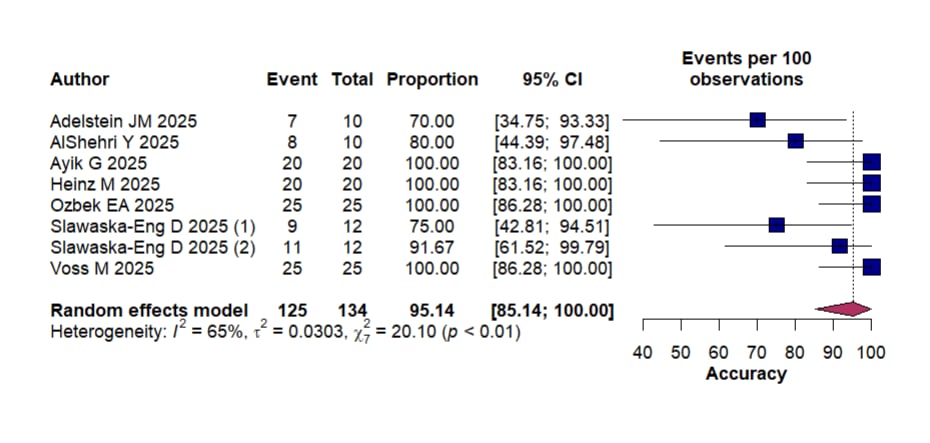

Supplement: Supplementary file 1 — Supporting information. [file KSA-34-2236-s002.JPG]

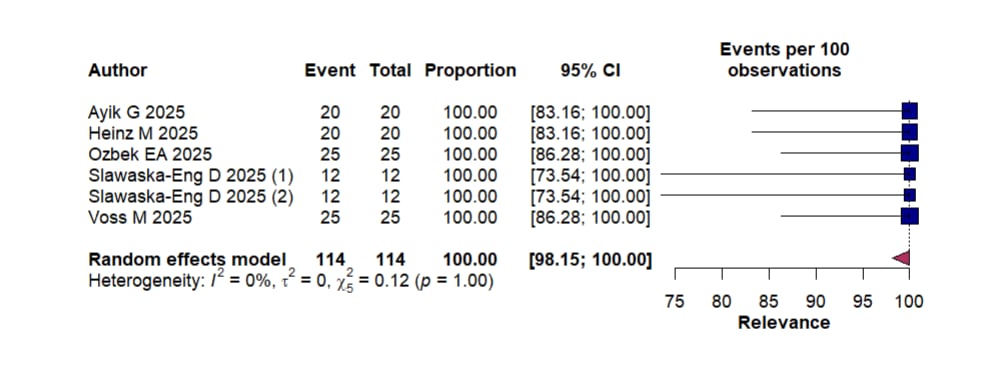

Supplement: Supplementary file 2 — Supporting information. [file KSA-34-2236-s011.JPG]

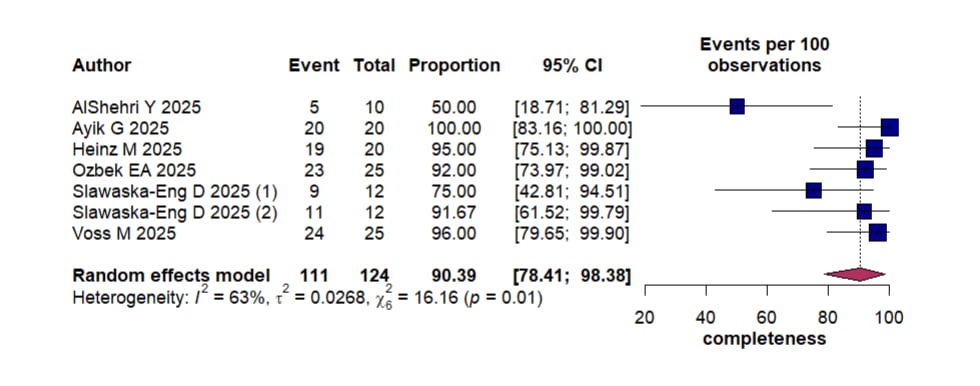

Supplement: Supplementary file 3 — Supporting information. [file KSA-34-2236-s001.JPG]

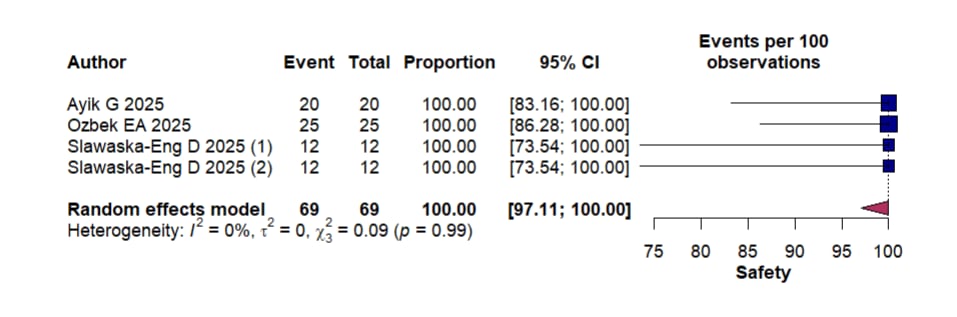

Supplement: Supplementary file 4 — Supporting information. [file KSA-34-2236-s014.JPG]

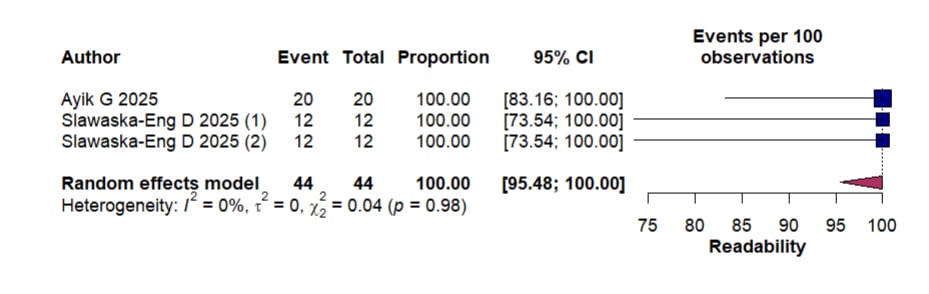

Supplement: Supplementary file 5 — Supporting information. [file KSA-34-2236-s004.JPG]

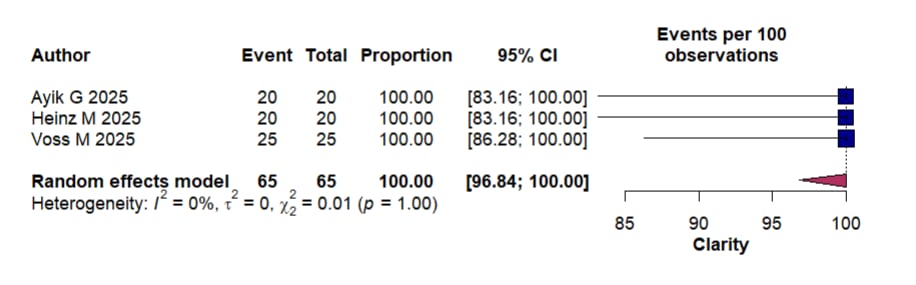

Supplement: Supplementary file 6 — Supporting information. [file KSA-34-2236-s009.JPG]
