# Supplementary material for: ChatGPT provides accurate and safe responses to patient questions on hip arthroscopy, while completeness remains variable: A systematic review and single‐arm meta‐analysis
Source: Knee Surg Sports Traumatol Arthrosc. 2026 Apr 14;34(6):2236–46. doi: 10.1002/ksa.70396 (PMC13266954; doi:10.1002/ksa.70396)
Supplement: Supplementary file 14 — Supporting information. [file KSA-34-2236-s012.docx]

**SUPPLEMENTARY APPENDIX**

**Supplementary Figure 1: Contour-enhanced funnel plot for accuracy.** The funnel plot showed no marked asymmetry, and no small-study effects were observed.

**Supplementary Figure 2: Contour-enhanced funnel plot for relevance.** The distribution was symmetrical, and no small-study effects or publication bias were detectable.

**Supplementary Figure 3: Contour-enhanced funnel plot for completeness.** The plot showed a dispersed but overall symmetric distribution without clear evidence of small-study effects.

**Supplementary Figure 4: Contour-enhanced funnel plot for safety.** The plot was symmetrical; interpretation remains limited because only few studies contributed data.

**Supplementary Figure 5: Contour-enhanced funnel plot for readability.** Two studies were tightly clustered with symmetrical distribution and no indication of small-study effects.

**Supplementary Figure 6: Contour-enhanced funnel plot for clarity.** Three clustered studies showed a symmetrical distribution without signs of small-study effects.

**Supplementary Figure 7:** **Forest plot of accuracy proportions, based on a sensitivity analysis excluding Gültekin et al. [9].** Accuracy ranged from 70% to 100%, with a pooled value of 95.1% (95% CI 85.1–100.0). Heterogeneity was moderate (I² = 65%).

**Supplementary Figure 8:** **Forest plot of relevance proportions, based on a sensitivity analysis excluding Gültekin et al. [9].** All studies reported 100% relevance, resulting in a pooled estimate of 100% (95% CI 98.2–100.0) with no heterogeneity (I² = 0%).

**Supplementary Figure 9**: **Forest plot of completeness proportions, based on a sensitivity analysis excluding Gültekin et al. [9].** Completeness ranged widely from 50% to 100%, with a pooled value of 90.4% (95% CI 78.4–98.4). Heterogeneity was moderate (I² = 63%).

**Supplementary Figure 10:** **Forest plot of safety proportions, based on a sensitivity analysis excluding Gültekin et al. [9].** All studies reported 100% safety, producing a pooled value of 100% (95% CI 97.1–100.0), with no heterogeneity.

**Supplementary Figure 11:** **Forest plot of readability proportions, based on a sensitivity analysis excluding Gültekin et al. [9].** Both included studies reported 100% readability, with a pooled estimate of 100% (95% CI 95.5–100.0) and no heterogeneity.

**Supplementary Figure 12:** **Forest plot of clarity proportions, based on a sensitivity analysis excluding Gültekin et al. [9].** All studies reported 100% clarity, yielding a pooled value of 100% (95% CI 96.8–100.0) with no heterogeneity.

**Supplementary Table 1**: PRISMA 2020 checklist.
